# ﻿*Pseudobarbusouteniqua* sp. nov., a new redfin species (Teleostei, Cyprinidae) from the Cape Fold Ecoregion of South Africa

**DOI:** 10.3897/zookeys.1239.131064

**Published:** 2025-05-28

**Authors:** Fatah Zarei, Pedro H. N. Bragança, Paul H. Skelton, Albert Chakona

**Affiliations:** 1 NRF-South African Institute for Aquatic Biodiversity (NRF-SAIAB), P. Bag 1015, Makhanda 6140, South Africa NRF-South African Institute for Aquatic Biodiversity Makhanda South Africa; 2 Department of Ichthyology, American Museum of Natural History, Central Park West at 79th Street, New York, NY 10024, USA American Museum of Natural History New York United States of America; 3 Department of Ichthyology and Fisheries Science, Rhodes University, PO Box 94, Makhanda 6140, South Africa Rhodes University Makhanda South Africa

**Keywords:** Conservation, endemic hotspot, Forest lineage, minnows, systematics, threatened freshwater fish

## Abstract

Previous DNA-based studies identified four genetic lineages within *Pseudobarbusafer*: (i) the Mandela lineage confined to the Sundays, Swartkops, and Baakens river systems, (ii) the Krom lineage endemic to the Krom River system, (iii) the St Francis lineage occurring in the Gamtoos and adjacent river systems, and (iv) the Forest lineage occurring in several southern coastal river systems from the Tsitsikamma to the Klein Brak river system. Subsequent detailed morphological evaluation provided a redescription of *P.afer* s.s. (Mandela lineage), supported revalidation of *P.senticeps* (Krom lineage) and description of a new species, *P.swartzi* (St Francis lineage). The present study builds on these earlier findings and provides a formal description of the Forest lineage as a new species for science, *Pseudobarbusouteniqua***sp. nov.** The new species differs from the aforementioned congeners by the conspicuous pigmentation on the centre of the scales which gives it a distinctive speckled hen pattern. The new species thus closely resembles the small-scale redfin, *P.asper*, in colour pattern, but it is readily separated from this species by genetic characters and fewer number of scales in predorsal region (16–17, mode 16 vs 18–26, mode 20–23) and around the caudal peduncle (14–15, mode 15 vs 16–22, mode 18–20). A revised key for the single-barbeled redfins is presented.

## ﻿Introduction

The genus *Pseudobarbus* Smith, 1841 represents a distinct clade of red-finned tetraploid smiliogastrine minnows endemic to the Cape Fold Mountain streams, from the Sundays River in the east to the Olifants River in the west (Skelton 2001; Ellender et al. 2017; Chakona et al. 2022). Minnows from the Cape with characteristic ‘redfins’ were first recognised as such by Barnard (1943) and then more clearly as a group by Jubb (1965, 1967). Although *Pseudobarbus* was first established by Smith (1841) as a subgenus of *Barbus* Daudin, 1805, only approximately 150 years later Skelton (1988) raised *Pseudobarbus* to generic status to accommodate this monophyletic group of tetraploid minnows. *Pseudobarbus* species are characterised by presence of bright red scarlet patches on the base of fins, possession of a flexible primary dorsal spine and development of prominent nuptial tubercles in breeding males. In his revision, Skelton (1988) recognised seven species [*P.burchelli* Smith, 1841, *P.burgi* (Boulenger, 1911), *P.phlegethon* (Barnard, 1938), *P.tenuis* (Barnard, 1938), *P.afer* (Peters, 1864), *P.asper* (Boulenger, 1911), and *P.quathlambae* (Barnard, 1938)], six of which were confined to the Cape Fold Ecoregion (CFE) and *P.quathlambae* endemic to the Highlands of Lesotho and the Mkomazana River, an escarpment headwater stream in Kwa-Zulu-Natal. Over two decades later, three new species, *P.skeltoni* Chakona & Swartz, 2013, *P.verloreni* Chakona, Swartz & Skelton, 2014 and *P.swartzi* Chakona & Skelton, 2017 were described, and *P.senticeps* (Smith, 1936) was reinstated as a valid species (Chakona and Skelton 2017). Molecular evidence from previous studies, however, showed that there are at least six additional lineages of *Pseudobarbus* that await formal description (Swartz et al. 2004, 2007, 2009; Chakona et al. 2013a). More recently, Skelton (2024) revalidated *P.vulnerata* (Castelnau, 1861) from the Breede and adjacent river systems and Zarei et al. (2025) described a new species, *P.kubhekai* Zarei, Mathebula & Chakona, 2025, from the Mzimkhulu River system in KwaZulu Natal. These revisions have increased the number of species in the genus *Pseudobarbus* from seven in Skelton (2001) to 13 currently recognised species (Skelton 2024; Zarei et al. 2025).

Skelton’s (1988) treatment of *Pseudobarbusafer* (Peters, 1864) considered the species to inhabit the rivers of the Eastern Cape from the Sundays in the east to the Tsitsikamma in the west, as well as a cluster of streams further west draining the southern slopes of the Outeniqua Mountains around the towns of Plettenberg Bay and Knysna (Chakona and Skelton 2017: fig. 1). Previously, Barnard (1943) and Jubb (1965, 1967) considered these latter south coastal populations to be a variant of *P.asper*. Barnard’s consideration was based in part on the living colours of the fishes, as he wrote “But when alive they had a beautiful warm brown or golden brown appearance, in marked contrast to the usual silvery grey colour of the typical form” (Barnard 1943: 200). Barnard pointed out that the coastal streams were highly acidic (pH 4–4.5) whereas the streams of the Gourits River system where the typical form occurs are alkaline (pH 8–9).

Swartz et al. (2007, 2009) identified four genetic lineages within populations that were previously assigned to *P.afer* sensu lato based on the analysis of mitochondrial cytochrome *b* (cyt *b*) and Control Region sequences. The ‘Krom’ lineage was confined to the Krom River system, the ‘St. Francis’ lineage occurred in the Gamtoos and adjacent river systems, the ‘Mandela’ lineage occurred in the Swartkops and Sundays river systems and a separate ‘Forest’ lineage, sister to the fiery redfin, *P.phlegethon*, occurred in the coastal rivers of the Outeniqua and Tsitsikamma mountain ranges from the Tsitsikamma, Bloukrans (east) to the Klein Brak River system (west). Chakona and Skelton (2017) examined specimens of the Krom, St. Francis and Mandela lineages and found them to be morphologically and therefore diagnostically distinct. Based on this evidence, Chakona and Skelton (2017) reinstated *P.senticeps* as a valid species and conspecific to the ‘Krom’ lineage, described a new species, *P.swartzi* for the ‘St. Francis’ lineage, and redescribed *P.afer* sensu stricto for the ‘Mandela’ lineage. They accepted that the ‘Forest’ lineage does not belong to *P.afer* complex as phylogenetically it was most closely related to *P.phlegethon* from the Olifants-Doring system on the west coast of South Africa (Swartz et al. 2007, 2009).

In the present study, we examined specimens collected from multiple populations of the ‘Forest’ lineage, providing evidence that they are morphologically distinct from all known congeners. Based on these findings, we describe the ‘Forest’ lineage as a new species, *Pseudobarbusouteniqua* sp. nov.

## ﻿Materials and methods

Institutional abbreviations follow Sabaj (2023) and are listed at https://asih.org/. Description of *Pseudobarbusouteniqua* sp. nov. is based on 15 specimens (holotype and paratypes) that were collected from the Klein Brak River system during a survey conducted in February 2024. The fishes were collected using a seine net (3 m length, 1.5 m width, 3 mm mesh size). Approximately 180–200 individuals were collected. A sub-sample (up to 20 specimens) of the collected specimens was anaesthetised with clove oil, and selected specimens were photographed in the field to capture the live colouration pattern. The rest of the specimens were returned to the river alive. Tissue samples were taken for DNA analysis and preserved in 96% ethanol and subsequently kept at -80 °C until DNA extraction. The source specimens were initially fixed in 10% formalin for one week, after which they were transferred to 70% ethanol for long-term storage. The DNA tissue samples, as well as the holotype (SAIAB 237307) and 10 paratypes (SAIAB 246084) were deposited into the Fish Collection Facility at the NRF-South African Institute for Aquatic Biodiversity (**NRF-SAIAB**) in Makhanda (formerly Grahamstown). Additional paratypes were deposited in the Natural History Museum, London, UK. (**NHMUK**: *n* = 2; NHMUK 2024.11.26.1-2) and the American Museum of Natural History, New York, USA. (**AMNH**: *n* = 2; AMNH 281979).

### ﻿Molecular data

The mitochondrial cytochrome *b* gene (cyt *b*) for four specimens of *Pseudobarbusouteniqua* sp. nov. from the Klein Brak River (SAIAB SB11791 to SB11794) were sequenced to assign hologenetype and paragenetypes following Chakrabarty (2010). Two new cyt *b* sequences were also generated for *P.phlegethon* from the Rondegat River in the Olifants River system. The new sequences were incorporated into the genetic data from Swartz (2005), Chakona and Swartz (2013), Machordom and Doadrio (2001), Tsigenopoulos et al. (2002), Chakona et al. (2013a, 2014), and Zarei et al. (2025) to show the phylogenetic position and distinctiveness of the hologenetype and the paragenetypes in relation to all known species of *Pseudobarbus* (Table 1). Methods of DNA extraction, amplification, sequencing, and analysis followed Swartz et al. (2009) and Chakona and Swartz (2013). The hologenetype and paragenetype sequences were deposited in GenBank for future reference (GenBank accession number are given below) following the definitions of Chakrabarty (2010).

**Table 1.** List of *Pseudobarbus* specimens used in the phylogenetic analyses (cyt *b* and Control Region), including GenBank accession numbers.

	cyt *b*			Control Region		
	Locality	N	GenBank No.	Locality	N	GenBank No.
*P.afer*	Sundays and Swartkops rivers	3	KY472280–KY472281, KY472285	Sundays and Swartkops rivers	10	EF376224–EF376233
*P.asper*	Vlei, Gourits River	1	AF287451	Gourits River	8	EF376255, EF376258–EF376263, EF376265
	Groot, Gamtoos River	1	AF180850	Groot at Steytlerville, Gamtoos River	3	EF376256–EF376257, EF376264
*P.burchelli*	Tradouw River	1	KF222702	Tradouw River	1	EU341796
	Heuningnes River	1	EU341732	–	–	–
*P.burgi*	Tulbagh, Berg River	1	AF180849	–	–	–
*P.kubhekai*	Mzimkhulu River	1	PQ367263	Mzimkhulu River	2	OP413900, OP413906
*P.outeniqua* sp. nov.	Klein Brak River	4	PQ653963–PQ653966*	Klein Brak River	4	EF376194–EF376197
	–	–	–	Tsitsikamma River	2	EF376215–EF376216
	–	–	–	Lakes Region rivers: Kaaimans, Touws, Duiwe and Karatara	7	EF376198–EF376204
	–	–	–	Plettenberg Bay rivers: Bitou, Keurbooms, Groot and Bloukrans	10	EF376205–EF376214
*P.phlegethon*	Lower Rondegat, Olifants River	2	PQ653967–PQ653968*	Olifants-Doring River system	11	EF376244–EF376254
	Noordhoeks	1	AF287452	–	–	–
*P.quathlambae*	Eastern Lesotho	2	AY791824–AY791825	Eastern Lesotho	1	AY791773
	Central Lesotho	1	AY791827	Central Lesotho	1	AY791787
*P.senticeps*	Krom River	2	KY472273–KY472274	Krom River	7	EF376217–EF376223
*P.skeltoni*	Riviersonderend, Breede River	1	KF222579	–	–	–
*P.swartzi*	Gamtoos River	3	KY472266, KY472272, KY472275	Gamtoos, Kabeljous and Swart rivers	10	EF376234–EF376243
*P.tenuis*	Vlei, Gourits River	2	AF287453–AF287454	Gourits River	25	EF376273– EF376290
	–	–	–	Keurbooms and Bitou rivers	9	EF376291–EF376299
*P.verloreni*	Verlorenvlei River	1	KM366106	–	–	–
*P.vulnerata*		1	KF222754	–	–	–

* Data from this study. N, number of sequences.

The saturation test by Xia et al. (2003) was performed in DAMBE 7 (Xia 2018) and tested for nucleotide substitution saturation in the cyt *b* dataset. The best-fit nucleotide substitution model, GTR+G+I, was estimated based on the Bayesian information criterion (BIC) in jModelTest 2.1.3 (Darriba et al. 2012). For phylogenetic reconstruction, the Bayesian Inference (BI) method was run based on four simultaneous runs of four Markov chains for 20,000,000 generations, with tree sampling every 1,000 generations and a burn-in of 20% of the initial trees in MrBayes 3.2.6 (Ronquist et al. 2012). A sequence of *Sedercypriscalidus* (Barnard, 1938) (GenBank accession number: AF287423) was used as outgroup. Average sequence divergence values between the studied species were estimated using the Kimura-2-Parameter (K2P) model in MEGA 7.0 (Kumar et al. 2016). Four methods of molecular species delimitation were applied on the cyt *b* dataset: (i) Statistical Parsimony (SP) analysis based on a 95% connection probability threshold in TCS 1.21 (Clement et al. 2000); (ii) Assemble Species by Automatic Partitioning (ASAP) on its server (https://bioinfo.mnhn.fr/abi/public/asap/asapweb.html) using the Kimura (K80) ts/tv (= 0.2) (Puillandre et al. 2021); (iii) Bayesian Poisson Tree Process (bPTP) on its server (https://species.h-its.org/ptp/) using a Bayesian topology of the sequences from MrBayes as input tree and run under default settings (Zhang et al. 2013); and (iv) the Generalized Mixed Yule Coalescent (bGMYC) on its server (https://species.h-its.org/gmyc/) and run under default settings (Fujisawa and Barraclough 2013) using an ultrametric tree from BEAST 1.8.2 (Drummond et al. 2012).

The molecular analysis also included a comprehensive phylogenetic assessment of 108 previously published mitochondrial Control Region sequences (608 bp) from Swartz (2005) and Swartz et al. (2007, 2023) representing all single-barbeled *Pseudobarbus* species (Table 1). This analysis aimed to characterise the distinctiveness and phylogenetic positioning of *P.outeniqua* sp. nov. and to examine its genetic structure within its geographic distribution. Phylogenetic analysis was conducted using the T92+G+I nucleotide substitution model, identified as the best fit, and the maximum likelihood (ML) method in MEGA. A sequence of *Pseudobarbusburchelli* (GenBank accession number: EU341796; Swartz et al. 2014), a double-barbeled species, was used as outgroup.

### ﻿Morphological data

Meristic and morphometric characters were examined following Hubbs and Lagler (1958), Skelton (1988), Chakona and Swartz (2013), and Chakona and Skelton (2017). The characters considered for each specimen in the present study include 22 morphometric measurements and 12 meristic counts. Measurements were taken with digital callipers to the nearest 0.1 mm. We compared morphological and meristic differences among all single-barbeled redfins using data from Skelton (1980, 1988), Chakona and Skelton (2017) and Zarei et al. (2025). Morphometric measurements and meristic counts were also taken from topotypic specimens of *P.asper* (Groot River at Steytlerville, Gamtoos system and Le Roux River, Gourits system). A total of 27 specimens of the Forest lineage were measured for the present study. Descriptive statistics were computed using IBM SPSS Statistics 27. Following Randall (2001), morphometric data are given as a ratio in the text and as percentages of standard length (**SL**) and head length (**HL**) in the table. Osteology was checked based on X-ray images prepared with an Inspex 20i Digital Radiography System (Kodex Inc., New Jersey, U.S.A.) at NRF-SAIAB.

## ﻿Results

### ﻿Molecular data

**Figure 1.**
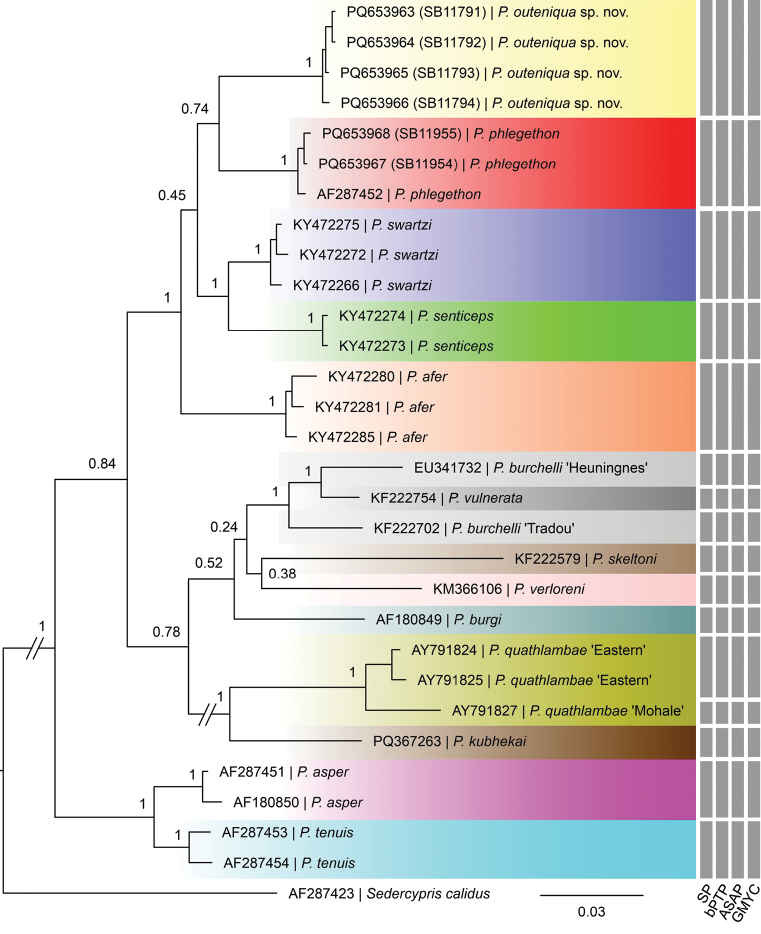
Bayesian Inference (BI) phylogeny of the genus *Pseudobarbus* reconstructed based on cyt *b* sequences, showing phylogenetic placement of *P.outeniqua* sp. nov. Grey bars to the right indicate species delimitation results.

We compared the cyt *b* sequences of four *P.outeniqua* sp. nov. specimens with 25 other *Pseudobarbus* sequences, representing all 13 currently described species in the genus. The substitution saturation test of Xia et al. (2003) for all three codon positions showed that the Index of Substitution Saturation (*Iss*) was significantly lower (P < 0.0001) than the critical Index of Substitution Saturation (*Iss. c*) for symmetrical and asymmetrical topologies, indicating that the cyt *b* sequences are applicable for phylogenetic analysis. The Bayesian phylogeny in Fig. 1 shows the phylogenetic placement of *P.outeniqua* sp. nov. based on cyt *b* sequences. The four studied individuals of *P.outeniqua* sp. nov. defined a distinct lineage sister to *P.phlegethon*. The single-barbeled species, *P.outeniqua* sp. nov., *P.phlegethon*, *P.swartzi*, *P.senticeps* and *P.afer* formed a monophyletic clade with high posterior probability (PP) support. The range of K2P genetic distance (%) between *P.outeniqua* sp. nov. and the other *Pseudobarbus* species is 4.7–12.7% (Table 2). The new species shows the lowest genetic distance value to *P.swartzi* (4.7%) and the highest genetic distance to *P.quathlambae* (12.7%). The four conceptually different molecular species delimitation methods (i.e., SP, bPTP, ASAP and GMYC) congruently revealed 16 putative species in the cyt *b* dataset comprising 29 sequences (Fig. 1). All molecular species delimitation methods identified the *P.outeniqua* sp. nov. lineage as a distinct taxonomic entity.

**Table 2.** Average K2P genetic distance (%) between *Pseudobarbus* spp. based on cyt *b*.

		1	2	3	4	5	6	7	8	9	10	11	12	13
1	*P.outeniqua* sp. nov.													
2	*P.burchelli*	6.6												
3	*P.skeltoni*	7.4	7.6											
4	*P.burgi*	6.9	5.6	9.1										
5	*P.verloreni*	7.6	6.0	8.7	6.9									
6	*P.asper*	7.2	7.4	11.1	7.4	8.3								
7	*P.tenuis*	8.1	7.2	10.9	7.8	6.9	3.2							
8	*P.afer*	6.5	5.8	8.9	6.9	7.2	7.8	6.5						
9	*P.phlegethon*	5.0	6.4	9.6	6.6	8.8	7.4	7.5	4.7					
10	*P.senticeps*	4.9	6.5	8.8	6.9	6.6	7.2	6.5	5.2	5.1				
11	*P.swartzi*	4.7	5.3	8.1	6.0	6.9	6.6	5.5	4.5	4.4	2.7			
12	*P.kubhekai*	11.6	10.2	13.7	10.8	10.9	13.1	11.3	12.6	13.0	12.0	12.3		
13	*P.quathlambae*	12.7	11.0	14.0	13.3	13.1	13.5	13.1	12.9	13.2	12.5	11.9	6.9	
14	*P.vulnerata*	7.0	3.2	7.7	6.1	6.6	8.0	7.6	6.5	7.0	7.2	6.0	10.6	11.1

**Figure 2.**
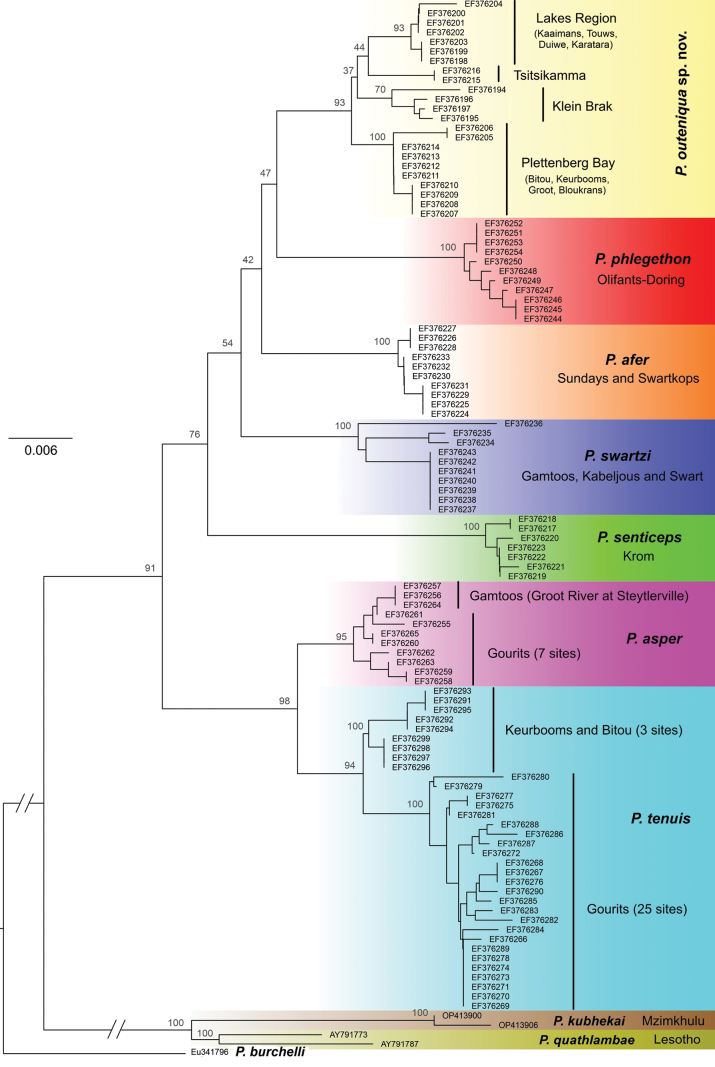
Maximum likelihood (ML) phylogeny of single-barbeled *Pseudobarbus* species based on mtDNA Control Region, showing phylogenetic placement and genetic structuring of *P.outeniqua* sp. nov.

The ML phylogeny of all single-barbeled *Pseudobarbus* species, based on Control Region sequences, reveals that specimens of *P.outeniqua* sp. nov. from the Klein Brak, Tsitsikamma, Kaaimans, Touws, Duiwe, Karatara, Bitou, Keurbooms, Groot, and Bloukrans rivers form a monophyletic clade (Fig. 2). This clade is sister to *P.phlegethon*. Consistent with Swartz et al. (2007), *P.outeniqua* sp. nov. comprises four allopatric haplogroups: (i) Klein Brak, (ii) Tsitsikamma, (iii) Lakes Region rivers, including Kaaimans, Touws, Duiwe, and Karatara, and (iv) Plettenberg Bay rivers, including Bitou, Keurbooms, Groot, and Bloukrans.

### ﻿Morphological data

#### ﻿Taxonomy

##### ﻿ Pseudobarbus outeniqua sp. nov.

Taxon classificationAnimaliaCypriniformesCyprinidae

277FE934-02B7-5C37-BECB-7AE257A9D3AD

https://zoobank.org/AD1FFBD5-7775-4893-951A-02E376F5E46E

Figs 3 , 4

Barbus asper : Barnard (1943); Jubb (1965, 1967).
Pseudobarbus afer : Skelton (1988).
Pseudobarbus sp. ‘afer Forest’: Swartz et al. (2007, 2009); Skelton (2024).

###### Proposed common names.

Forest redfin (English), Wildernis rooivlerkie (Afrikaans).

###### Type material.

***Holotype*.** • SAIAB 237307 (tag number FZ03), male, 67.6 mm SL, Kouma River at Willem’s Farm, Klein Brak River system, South Africa, -33.95261111, 21.97691667, collected by A. Chakona, N. Mazungula and X. Mathebula, 25 February 2024. Hologenetype: • SAIAB SB11793, GenBank number: PQ653965. ***Paratypes*** (*n* = 14). • SAIAB 246084 (tag numbers FZ01–FZ02, FZ04, FZ06–FZ07, FZ10– FZ11, FZ13–FZ15), 10 unsexed, 46.6–83.2 mm SL, same locality information and collectors as holotype. • BMNH 2024.11.26.1-2 (tag numbers FZ09 & FZ12), 2 unsexed, 67.2–80.3 mm SL, same locality information and collectors as holotype. • AMNH 281979 (tag numbers FZ05 & FZ08), 2 unsexed, 77.3–80.4 mm SL, same locality information and collectors as holotype. Paragenetypes: SAIAB SB11791–SB11792, SB11794, 3 specimens, GenBank number: PQ653963–PQ653964, PQ653966.

###### Additional non-type materials

(*n* = 29). • SAIAB 246101, 17 unsexed, 29.1–57.8 mm SL, Touws River, South Africa, -33.94672778, 22.61263611, collected by A. Chakona, P.H. Skelton and P. Bragança, 21 February 2023. • SAIAB 128708, 5 unsexed, 50.1–67.4 mm SL, Causeway at Kruisvallei/George, Keurbooms, South Africa, -33.812, 23.17472, collected by J. Olivier and S. Thorne, 01 March 1983. • SAIAB 200541, 2 unsexed, 74.9–86.9 mm SL, Kwaai River, Keurbooms, South Africa, -33.82, 23.17972, collected by E. Swartz, 11 April 2000. • SAIAB 128186, 3 unsexed, 48.6–52.6 mm SL, Kaapsedrif, Tsitsikamma, South Africa, -34.16, 24.4, collected by A.H. Bok and M. King, 12 May 1982. • SAIAB 64260, 1 unsexed, 68.0 mm SL, Tsitsikamma National Park, Groot River, South Africa, collected by I.A. Russel, 26 February 2001. SAIAB 122981, 1 unsexed, 47.7 mm SL, Palmietvlei road at Kaapsedrif, Tsitsikamma, South Africa, -34.05, 24.4, collected by D. Heard, 28 October 1976.

###### Diagnosis.

*Pseudobarbusouteniqua* sp. nov. is diagnosed among all currently recognised congeners by the following combination of character states: mouth with one pair of barbels; barbel length 1.0–1.9 times orbit diameter, reaching vertical through posterior edge of eye; pigmentation distinct, with scale centres darkly pigmented, giving the fish an overall speckled appearance, speckling less conspicuous or absent ventrally; presence of a distinct dark mid-lateral band, with a broader anterior half and a narrower posterior half which ends in form of a large triangular mark at the base of the caudal fin; lack of dark spots, dashes, stripes or wavy lines on back and mid-dorsal; scales moderate sized, 35–37 in lateral line series, 14–15 (mode 15) around caudal peduncle, and 16–17 (mode 16) on predorsal region. Detailed comparison of the new species with the other congeners is presented below.

###### Description.

All morphometric values in the text are presented as holotype first and paratypes, if different, in parentheses. The following description is based on holotype and paratypes from the Klein Brak River system (westernmost population); data for additional non-type specimens from the Keurbooms (central population) and Tsitsikamma (easternmost population) rivers are given in Table 3.

**Table 3.** Morphometric and meristic data for *Pseudobarbusouteniqua* sp. nov. and its related congeneric species.

		*P.outeniqua* sp. nov.			*P.phlegethon*	*P.afer*	*P.senticeps*	*P.swartzi*	*P.asper*
		holotype	paratypes (*n* = 14)	non-type specimens (*n* = 12)	topotypes (*n* = 22)	syntypes + other specimens including topotypes (*n* = 71)*	holotype + topotypes (*n* = 30)*	holotype + other specimens including paratypes (*n* = 64)*	topotypes (Groot River at Steytlerville and Le Roux River) (*n* = 16)
SL	Standard length (mm)	67.6	46.6–83.2	47.7–86.9	45.5–61.8	43.0–82.0	45.0–79.3	46.5–75.2	47.5–76.4
HL	Head length (mm)	19.5	13.0–22.8	13.8–23.7	11.4–16.4	11.7–22.5	12.8–21.1	12.8–21.2	12.4–21.0
% **of SL**									
HL	Head length	28.9	27.3–28.9	27.3–30.7	24.4–26.6	24.1–30.0	26.1–29.6	26.6–30.0	22.7–28.7
PDL	Pre-dorsal length	56.3	54.0–56.3	54.2–56.5	53.0–56.5	49.6–55.5	49.3–55.0	53.3–56.6	51.4–56.7
DB	Dorsal fin base	13.6	11.5–13.6	12.5–14.3	12.4–14.2	11.0–14.4	11.0–14.9	10.8–13.4	12.4–14.4
DH	Dorsal fin height	24.1	21.2–24.0	22.3–26.3	21.3–25.0	20.4–25.2	20.8–25.4	23.5–27.8	17.3–26.4
AfB	Anal fin base	11.1	9.7–11.3	9.3–11.7	9.6–11.5	9.0–11.4	9.8–11.9	8.2–12.2	9.8–11.5
PP	Pectoral to pelvic fin length	23.1	21.6–26.2	22.4–25.0	25.1–30.0	20.9–27.0	21.3–25.7	19.3–26.9	20.9–26.5
PA	Pelvic to anal fin length	12.3	11.5–15.4	12.4–14.9	13.1–16.1	13.1–18.1	12.7–17.5	13.1–17.4	12.0–17.3
BD	Body depth	27.6	23.7–27.6	25.5–31.2	23.9–27.3	22.5–31.6	22.9–28.7	22.6–25.9	23.7–28.6
BW	Body width	17.0	14.8–18.1	15.3–19.4	14.5–18.1	10.8–20.2	13.9–19.3	14.6–17.7	12.6–18.6
CPL	Caudal peduncle length	25.1	22.8–25.5	22.5–24.9	22.2–26.1	23.0–27.6	22.5–26.2	22.2–25.4	23.6–26.9
% **of HL**									
HD	Head depth	64.4	64.4–73.1	65.3–72.6	65.7–76.3	61.8–78.2	65.3–74.3	63.7–71.6	65.7–84.2
IO	Inter-obit	33.4	32.0–34.8	31.1–34.7	33.0–37.4	25.2–34.8	27.1–33.0	25.7–31.2	33.0–40.4
S	Snout length	36.6	31.4–37.3	28.1–34.5	29.3–33.5	24.0–39.0	29.8–37.9	28.9–34.0	30.4–40.9
PO	Post orbit	44.0	43.2–46.5	44.0–50.9	41.2–48.9	42.2–51.2	44.2–52.7	44.6–50.7	43.1–60.1
PB	Posterior barbel length	30.4	22.2–40.0	21.7–34.1	2.4–15.3	12.1–27.2	26.0–37.0	26.7–39.9	11.1–21.9
OD	Orbit diameter	23.0	20.1–26.0	19.3–27.0	24.0–29.7	21.7–30.4	23.5–29.5	23.0–27.7	20.5–28.9
% **of CPL**									
CPD	Caudal peduncle depth	49.8	46.5–53.5	50.4–57.1	42.4–53.4	40.3–61.5	43.0–54.5	43.6–54.3	44.2–53.8
**Meristics**									
UdR	Unbranched dorsal fin rays	iii	iii	iii	iii	iv (ii–iv)	iv (iii–iv)	iii	iii
BdR	Branched dorsal fin rays	7	7 (6–7)	7	7 (6–7)	7 (6–7)	7	7	7
UaR	Unbranched anal fin rays	iii	iii	iii	iii	iii	iii	iii	iii
BaR	Branched anal fin rays	5	5	5	5	5	5	5	5
PecR	Pectoral fin rays	16	15–16 (15–17)	15–16	14 (12–15)	15 (13–17)	14 (13–15)	14–16	13–17
PelR	Pelvic fin rays	8	8	8	8 (7–8)	8 (8–9)	8 (8–9)	8 (7–8)	8
LL	Lateral line scales	37	35–37	35 (35–38)	35–37 (35–38)	32 (29–35)	29 (25–30)	36 (35–37)	37–44
LD	Lateral line to dorsal fin scale rows	6	6	6 (5–6)	5	5 (4–6)	5 (4–5)	6 (6–7)	6–8
LP	Lateral line to pelvic fin scale rows	5	4 (4–5)	4 (4–5)	4 (4–5)	4 (3–5)	4 (3–4)	5 (4–5)	5–8
LA	Lateral line to anal fin scale rows	5	5 (4–5)	5 (4–5)	4 (4–5)	4 (3–5)	3 (3–4)	5	5–7
CP	Caudal peduncle scale rows	15	15 (14–15)	14 (14–15)	14 (13–15)	12 (12–16)	12 (10–12)	16 (15–16)	18–20
PDS	Predorsal scale rows	17	16 (16–17)	16 (16–17)	16 (16–18)	15 (13–16)	15 (12–15)	17–18 (16–20)	18–21
TV	Total vertebrae	37	36 (36–38)**		36 (35–36)	37 (36–39)	37 (35–38)	37 (37–38)	35–38
PcV	Precaudal vertebrae	20	19 (19–20)**		20 (19–20)	19 (18–20)	19 (18–19)	20 (19–20)	17–20
CV	Caudal vertebrae	17	17 (16–18)**		17 (16–17)	18 (17–19)	18 (16–18)	18 (17–18)	16–20
PdV	Predorsal vertebrae	12	12 (12–13)**		13 (12–13)	12 (11–13)	12 (11–13)	13 (12–13)	11–13

* Data from Chakona and Skelton (2017). ** Counts based on radiographs of 14 paratypes.

***General morphology*.** Body proportions and meristics are given in Table 3. Body moderately elongate, fusiform, with dorsal profile generally more convex than ventral profile, its depth at dorsal fin origin (deepest) 3.6 (3.7–4.2) in SL, body laterally compressed. Caudal peduncle shallow, its depth 0.5 times caudal-peduncle length. Head large, length 3.5 (3.5–3.7) in SL, depressed, depth 5.4 (4.9–5.5) in SL and 0.7 (0.7–0.8) times body depth. Postorbital profile steep. Snout blunt, short, oblique, convex, longer than eye, length 1.6 (1.2–1.8) times eye diameter and 2.7 (2.7–3.1) in head length. Eyes large, diameter 4.4 (3.9–5.0) in head length, and dorsolateral, not extending above dorsal profile, located closer to tip of snout than posterior margin of operculum. Interorbital wide and flat, width 1.5 (1.3–1.7) times eye diameter. Mouth sub-terminal, sickle shaped, its corner reaching vertical through middle of nares. Mouth with a single pair of long maxillary barbels, barbel length 1.3 (1.0–1.9) times orbit diameter, reaching vertical through posterior edge of the eye.

***Tuberculation*.** Mature breeding males with conical tubercles on snout and top of head. Bilateral clusters of large tubercles (2–4 per cluster) present on snout. A row of large tubercles extends in an arc above each naris (3 tubercles) to the antero-dorsal edge of the orbit and then continues posteriorly (3 smaller tubercles) along the dorsal edge of each orbit. Anterior dorsal cluster includes a few small tubercles. Posterior dorsal cluster includes scattered smaller tubercles, progressively become smaller posteriorly. No tubercles were observed on the surface of the fin rays or the free edge of the latero-dorsal scales in the examined specimens.

***Scales*.** LL 35–37 (holotype: 37; paratypes: 35:6, 36:3, 37:5), LD 6, LP 4–5 (holotype: 5; paratypes: 4:11, 5:3), LA 4–5 (holotype: 5; paratypes: 4:3, 5:11), CP 14–15 (holotype: 15; paratypes: 14:1, 15:13), PDS 16–17 (holotype 17; paratypes: 16:12, 17:2). Nape naked. Predorsal scales between posterior edge of head and dorsal fin origin embedded and smaller than flank scales. Triangular naked patch between gill cover and anterior base of pectoral fin present; ventral scales between pectoral fin origin and pelvic fin origin reduced and embedded. All scales cycloid.

***Fins*.** Dorsal fin rays iii/6–7 (holotype iii/7; paratypes: iii/6:1, iii/7:13); anal fin rays iii/5; pectoral fin rays 15–17 (holotype: 16; paratypes: 15:6, 16:7; 17:1); pelvic fin rays 8; caudal fin principal rays 10+9. Dorsal fin situated almost in the centre of the body (excluding caudal fin), origin slightly behind vertical through origin of pelvic fin, distal margin straight to slightly concave, tip of depressed dorsal fin almost reaches to vertical through posterior base of anal fin in mature males, reaches within two scales to vertical through posterior base of anal fin. Pectoral fins fan-shaped, larger in males than females, reaches and surpasses base of pelvic fin in males, reaches two scales to base of pelvic fin in females. Pelvic fin origin slightly in front of dorsal fin origin, tip of depressed pelvic fin does not reach anterior origin of anal fin, except in mature males. Anal fin distal margin almost straight to slightly convex, origin closer to anterior base of pelvic fin than caudal fin base. Caudal fin forked.

***Osteology*.** Vertebral column including Weberian apparatus and urostyle: total vertebrae 36–38 (holotype: 37; paratypes: 36:10, 37:3, 38:1), predorsal vertebrae 12–13 (holotype: 12; paratypes: 12:12, 13:2), precaudal vertebrae 19–20 (holotype: 20; paratypes: 19:9, 20:5), and caudal vertebrae 16–18 (holotype: 17; paratypes: 16:1, 17:12, 18:1).

**Figure 3.**
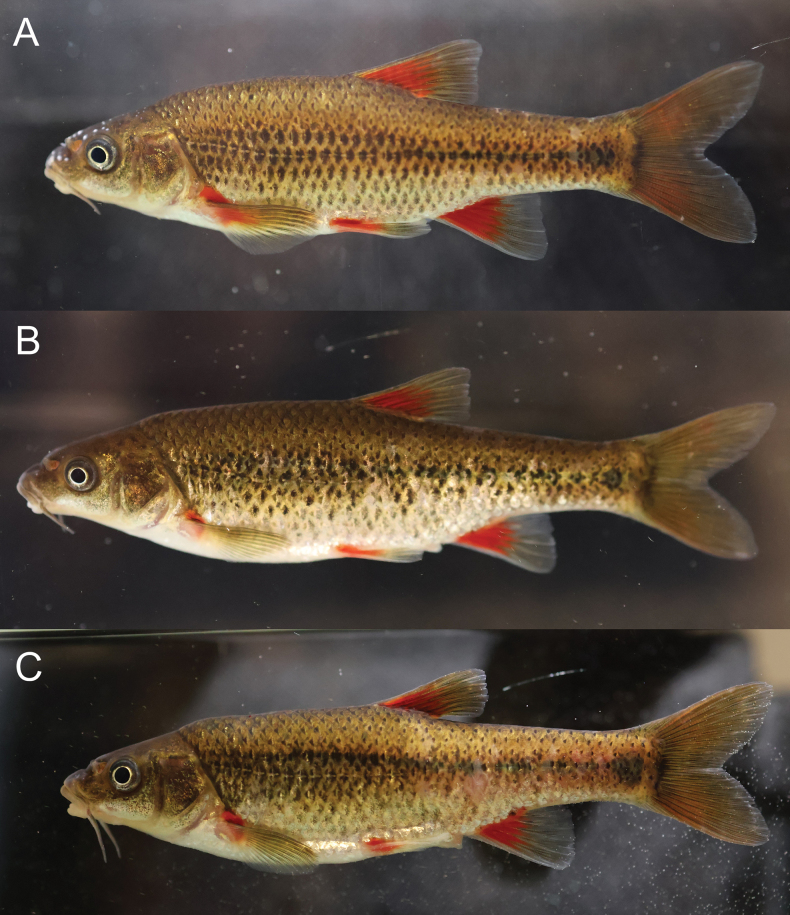
Live specimens of *Pseudobarbusouteniqua* sp. nov. from the Klein Brak River system **A** SAIAB 237307 (tag number FZ03), male, holotype, 67.6 mm SL**B** SAIAB 246084 (tag number FZ04), paratype, 80.6 mm SL**C** SAIAB 246084 (tag number FZ06), paratype, 77.9 mm SL.

***Colouration* (*live specimens*).** Refer to Fig. 3 for general live colouration. Body golden-tan laterally, becoming darker dorsally, and lighter to white ventrally. Scale centres darkly pigmented, giving an overall “speckled hen” appearance. Base of fins bright scarlet; caudal fin least affected by this colouration. Distinct dark mid-lateral band, covering three rows of scales, with a broaden anterior half from behind the head to vertical through anal-fin origin and a narrower posterior half to the base of the caudal fin, ends in form of a large triangular mark.

**Figure 4.**
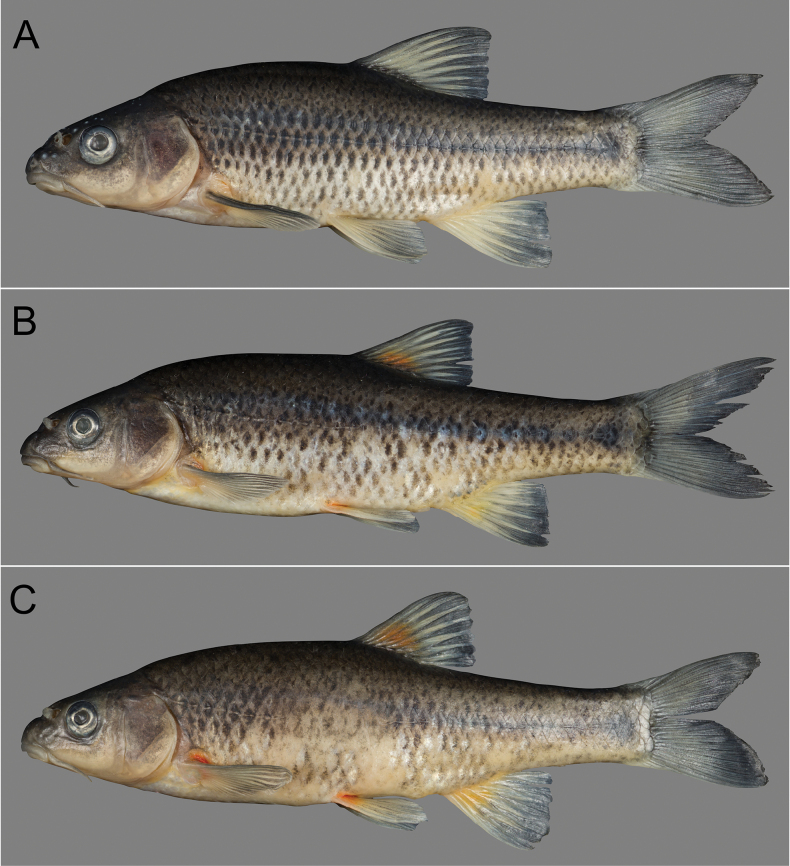
Preserved specimens of *Pseudobarbusouteniqua* sp. nov. from the Klein Brak River system **A** SAIAB 237307 (tag number FZ03), male, holotype, 67.6 mm SL**B** SAIAB 246084 (tag number FZ04), paratype, 80.6 mm SL**C** SAIAB 246084 (tag number FZ02), paratype, 83.2 mm SL.

***Colouration (preserved)*.** Background colour in alcohol preserved specimens after three months since collection, light brownish grey, becoming darker dorsally and lighter ventrally (Fig. 4). Scale centres darkly pigmented. Flanks with distinct dark mid-lateral band. The bright red pigmentation on base of fins first turns orange to yellow, then fades.

###### Distribution.

**Figure 5.**
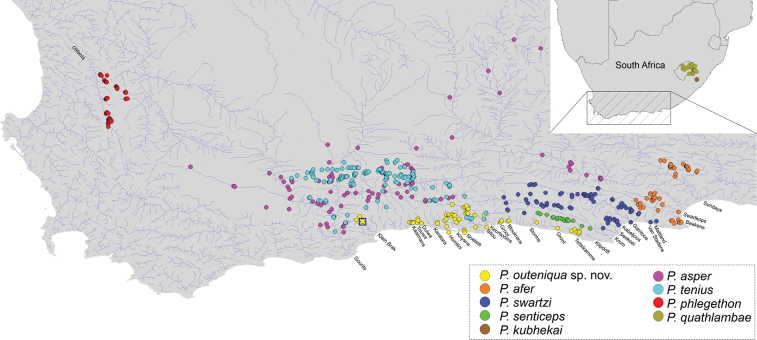
Distribution map of all single-barbeled *Pseudobarbus* species based on the SAIAB fish collection. The type locality of *Pseudobarbusouteniqua* sp. nov. at the Klein Brak River system is marked with a square.

*Pseudobarbusouteniqua* sp. nov. (referred to as the ‘Forest’ lineage by Swartz et al. 2007) is endemic to the Cape Fold Ecoregion of South Africa where it has a wide distribution range which includes a number of small isolated coastal catchments. The known distribution range extends from the Klein Brak River system in the Western Cape Province to the Tsitsikamma River system which is at the eastern end of the Western Cape Province (Fig. 5). There is very little information about the population sizes across this taxon’s distribution range, but ad hoc surveys indicate that the known populations are relatively abundant. The extent of alien fish invasion in the river systems where this taxon occurs is poorly documented. However, a trout farm exists within the Keurboom River catchment and that system has been extensively stocked with both brown and rainbow trout (Harrison 1966; Impson and Swartz 2011). *Tilapiasparrmanii* Smith, 1840 was also common in that system during our recent surveys.

###### Etymology.

The specific epithet *outeniqua* refers to the Outeniqua mountain range and highlights the species’ occurrence in streams draining its southern slopes within the southern Cape Fold Ecoregion.

###### Conservation status.

This species is threatened by invasive alien fish species, excessive water abstraction, increased sedimentation from forestry activities and pollution resulting from urban development and expansion. The spatial extent and severity of these impacts require further study. *Pseudobarbusouteniqua* sp. nov. occurs in isolated mountain tributaries. The species has an extent of occurrence (EOO) of 4035 km^2^ and an area of occupancy (AOO) of 168 km^2^. It is known from 11 distinct catchments and at least 16 locations [Klein Brak, Kaaimans, Touws (upper Touws and Duiwe River tributaries), Swartvlei (Karatara tributary), Goukamma/Homtini, Knysna (upper Knysna and Gouna tributaries), Keurbooms (upper Keurbooms, Bietou and Palmiet tributaries), Groot, Bloukrans and Tsitsikamma]. *Pseudobarbusouteniqua* sp. nov. was thus assessed as Near Threatened under criterion B1b(iii) + B2b(iii) (Chakona et al. 2022). The taxon is likely to be experiencing ongoing decline in quality of habitat due to water abstraction, agriculture, and urban development as well as impacts from forestry activities that has resulted in sedimentation of some of the streams. Invasive alien fish species likely pose a major threat to the extent of occurrence of this taxon but the degree of invasion and extent of impacts need to be assessed.

###### Habitat and ecology.

**Figure 6.**
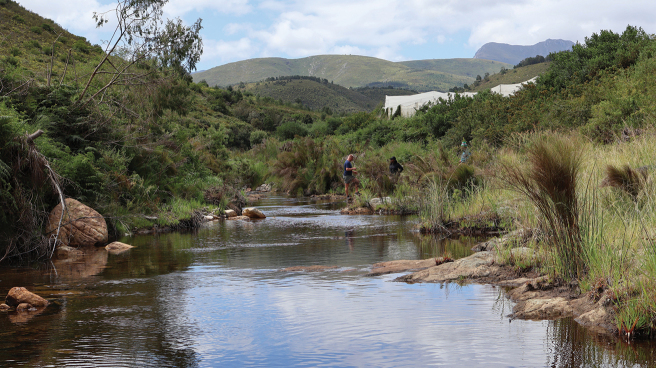
Habitat of *Pseudobarbusouteniqua* sp. nov., Kouma River at Willem’s Farm, Klein Brak River system.

This taxon occurs in dark peat-stained forest streams and prefers cover in a variety of microhabitats, ranging from pools with emergent vegetation (mainly palmiet), to runs and riffles with bed rock, boulders, and cobble substratum (Fig. 6). No information is available on the breeding ecology of this taxon, but it is likely to be similar to congeneric species that spawn after an increase in river flow during the rainy season, commencing in October and extending to February (Cambray 1994). Spawning generally occurs in riffle areas upstream of pools where eggs are deposited under mid-channel boulders. Once hatched, larvae drift from riffles into pool areas where they feed in the pelagic zone (Cambray 1994). Further research is required to improve our understanding of the ecology and biology of this taxon.

###### Comparative remarks.

*Pseudobarbusouteniqua* sp. nov. is easily distinguishable from *P.burchelli*, *P.burgi*, *P.skeltoni*, *P.verloreni*, and *P.vulnerata* by possession of a single pair of oral barbels (vs two pairs). The new species is also different form all single-barbeled *Pseudobarbus* species except *P.asper* in having an overall “speckled hen” colouration pattern.

*Pseudobarbusouteniqua* sp. nov. differs from *P.afer* in having longer barbels (22.2–40.0% HL and 1.0–1.9 times orbit diameter, reaching vertical through posterior edge of eye vs 12.1–27.2% HL and 0.4–1.1 times orbit diameter, barbels do not surpass the vertical through posterior margin of pupil), more scales in lateral line series (35–38, mode 35–37 vs 29–35, mode 32) and on predorsal region (16–17, mode 16 vs 13–16, mode 15), a mid-lateral band which terminates in a large triangular blotch at the base of the caudal fin (vs lack of a conspicuous blotch of pigment at the base of the caudal fin), and a different distributional range (coastal rivers of the Outeniqua and Tsitsikamma mountain ranges from Klein Brak east to Tsitsikamma vs the Sundays, Swartkops, and Baakens rivers, which discharge into the Algoa Bay; Fig. 5).

*Pseudobarbusouteniqua* sp. nov. further differs from *P.asper* in having a longer head (27.3–30.7 vs 24.7–28.2% SL), possession of fewer scales in lateral line series (35–37 vs 35–45, mode 37–40), on predorsal region (16–17, mode 16 vs 18–26, mode 20–23), around the caudal peduncle (14–15, mode 15 vs 16–22, mode 18–20), lateral line to dorsal fin origin (5–6, mode 6 vs 6–9, mode 7–8), lateral line to pelvic fin origin (4–5, mode 4 vs 5–8, mode 5–7), and lateral line to anal fin origin (4–5, mode 5 vs 5–8, mode 5–7), and a different distributional range (coastal rivers of the Outeniqua and Tsitsikamma mountain ranges from Klein Brak east to Tsitsikamma vs main branches of the Gourits and upper and middle reaches of Gamtoos system; Fig. 5) (morphological data for *P.asper* from widespread localities in the Gamtoos and Gourits systems, including 16 toptypic specimens in this study and 110 specimens from Skelton 1988).

*Pseudobarbusouteniqua* sp. nov. also differs from its closest relative, *P.phlegethon*, in having a longer head (27.3–28.9 vs 24.4–26.6% SL), longer barbels (22.2–40.0% HL and 1.0–1.9 times orbit diameter, reaching vertical through posterior edge of eye vs 2.4–15.3% HL and 0.1–0.6 times orbit diameter, not reaching vertical through posterior edge of eye), more pectoral fin rays (15–17, mode 15–16 vs 12–15, mode 14), lack of prominent black spots and patches on the body (vs presence), tuberculation of head in mature breeding males (presence of well-developed large to small conical tubercles on the snout and on top of the head vs head tubercles usually absent or poorly developed in some specimens), and a different distributional range (coastal rivers of the Outeniqua and Tsitsikamma mountain ranges from Klein Brak east to Tsitsikamma vs the Olifants-Doring River system on the west coast of South Africa; Fig. 5).

*Pseudobarbusouteniqua* sp. nov. is also easily distinguishable from *P.quathlambae* and *P.kubhekai* by having larger and fewer scales, 35–37 scales in lateral line series (vs scales very small, 51–72 scales in lateral line series), longer barbels (1.0–1.9 times orbit diameter, reaching vertical through posterior edge of eye vs less than orbit diameter, not reaching the vertical through the middle of pupil), tuberculation of head in mature breeding males (large to small conical tubercles on the snout and on top of the head vs numerous minute, conical tubercles on the snout, top of the head, operculum, and below the orbit), and a different distributional range (coastal rivers of the Outeniqua and Tsitsikamma mountain ranges from Klein Brak east to Tsitsikamma vs tributaries of the upper Orange River system in the Lesotho Highlands and the Mkhomazi and Mzimkhulu river systems in KwaZulu Natal; Fig. 5). It further differs from *P.quathlambae* by having fewer vertebrae (36–38, mode 36 vs 38–40, mode 39–40) and the absence of dark spots or wavy lines on the back (vs presence of bi-lateral series of discrete predorsal spots, dashes, or vermiculations). Skelton (1988) also showed that *P.afer* s.l. and *P.quathlambae* differ in the shape and number of pharyngeal teeth as well as gut length.

*Pseudobarbusouteniqua* sp. nov. also differs from *P.senticeps* in having more scales in the lateral line series (35–37 vs 25–30, mode 29), around the caudal peduncle (14–15, mode 15 vs 10–12, mode 12) and on the predorsal region (16–17, mode 16 vs 12–15, mode 15), and a different distributional range (coastal rivers of the Outeniqua and Tsitsikamma mountain ranges from Klein Brak east to Tsitsikamma vs the Krom River system; Fig. 5).

*Pseudobarbusouteniqua* sp. nov. also differs from *P.swartzi* in having a wider inter-obit (32.0–34.8% HL and 1.3–1.7 times eye diameter vs 25.7–31.2% HL and 1.0–1.3 times eye diameter), smaller modes for total vertebrae (36 vs 37), precaudal vertebrae (19 vs 20), caudal vertebrae (17 vs 18), and predorsal vertebrae (12 vs 13), a distinct mid-lateral band which terminates in a triangular blotch at the base of the caudal fin (vs mid-lateral band present but obscure, black blotch at the base of caudal fin inconspicuous), and a different distributional range (coastal rivers of the Outeniqua and Tsitsikamma mountain ranges from Klein Brak east to Tsitsikamma vs the Kougaberg, Baviaanskloofberg, and Elandsberg tributaries of the Kouga and Groot sub-catchments of the Gamtoos River system, and the Kabeljous and Swart river systems which discharge into the St Francis Bay; Fig. 5).

*Pseudobarbusouteniqua* sp. nov. also differs from *P.tenuis* by absence of a dark mid-dorsal stripe (vs presence of a dark mid-dorsal stripe which is sometimes interrupted to form a series of dashes), and having a broad mid-lateral band in front of vertical through anal-fin origin (vs mid-lateral band progressively becomes narrower anteriorly). *Pseudobarbusouteniqua* sp. nov. is deeper bodied than *P.tenuis* and differs in pharyngeal teeth and gut length characteristics (Skelton 1988). It has a different distributional range (coastal rivers of the Outeniqua and Tsitsikamma mountain ranges from Klein Brak east to Tsitsikamma vs main branches of the Gourits, Bitou, and Keurbooms; Fig. 5).

### ﻿Key to single-barbeled species of *Pseudobarbus*

**Table:** 

1	Scales small, > 50 in lateral line series	**2**
–	Scales moderately sized, < 45 in lateral line series	**3**
2	51–56 scales in lateral line series, absence of dark spots on back (Mzimkhulu River system)	***P.kubhekai***
–	60–72 scales in lateral line series, presence of 2–4 rows of dark spots on back (Upper Orange and Mkhomazi River systems)	***P.quathlambae***
3	Presence of prominent black spots and patches on the body (Olifants-Doring River system)	***P.phlegethon***
–	Absence of prominent black spots and patches on the body	**4**
4	25–30 scales in lateral line series, 10–12 circumpeduncular scales (Krom River system)	***P.senticeps***
–	29–39 scales in lateral line series, 12–16 circumpeduncular scales	**5**
5	Presence of mid-predorsal stripes and dashes (Gourits, Keurbooms and Bitou river systems)	***P.tenuis***
–	Absences of mid-predorsal stripes and dashes	**6**
6	29–35 scales in lateral line series, 13–16 predorsal scales (Sundays, Swartkops and Baakens rivers)	***P.afer***
–	35–38 scales in lateral line series, 16–20 predorsal scales	**7**
7	Colour pattern not “speckled hen” (river systems discharging into the St Francis Bay)	***P.swartzi***
–	Scale centres darkly pigmented, giving an overall “speckled hen” appearance	**8**
8	14–15 circumpeduncular and 16–17 predorsal scales (coastal rivers of the Outeniqua and Tsitsikamma mountain ranges)	***P.outeniqua***
–	16–22 circumpeduncular and 18–26 predorsal scales (Gourits and upper and middle reaches of the Gamtoos system)	***P.asper***

## ﻿Discussion

Despite a closer phylogenetic relationship to *Pseudobarbusphlegethon* from the Olifants-Doring River system on the west coast, *P.outeniqua* morphologically closely resembles its geographical neighbours from river systems on the south coast belonging to the *P.afer* group: *P.afer* s.s., *P.senticeps*, and *P.swartzi*. This close phylogenetic relationship between *P.outeniqua* and *P.phlegethon*, however, needs to be interpreted with caution as the relationship is not well-supported and the application of mtDNA data without nuclear data may not accurately reflect the underlying species tree (Avise 2000). The uniparental inheritance of mtDNA alleles preclude conclusive inference of the relationship between these two species. Considering the limitations of mitochondrial markers for phylogenetic reconstructions, analysis of combined nuclear, mitochondrial, and osteological dataset to unravel phyletic relationships and diversification patterns within *Pseudobarbus* remains of interest. There are ongoing efforts to sequence the nuclear genes of southern African cyprinids to enable us to unravel the evolutionary relationships and origins of the polyploid smiliogastrines in the tetraploid genera *Pseudobarbus*, *Sedercypris* Skelton, Swartz & Vreven, 2018, *Namaquacypris* Skelton, Swartz & Vreven, 2018, *Amatolacypris* Skelton, Swartz & Vreven, 2018 and *Cheilobarbus* Smith, 1841, endemic to this region.

Nevertheless, the pattern indicates that the two species have been in recent contact, with the Gourits River system being the logical pathway that could have facilitated the connection between the fish fauna from the westward and southward draining river systems. We hypothesise that representatives of one or both species or a common ancestor would have occurred in the Gourits River system in the recent past followed by subsequent extinction. Similar genealogical pattern has been recovered in other taxa including freshwater crabs (Daniels et al. 2006), and a galaxiid lineage (Chakona et al. 2013b) as well as some terrestrial insects with close affinities to riverine vegetation (Price et al. 2007).

The newly described species has a rather unusual wide distribution as it occurs across several river systems, from the Klein Brak on the west to the Tsitsikamma in the east (Swartz et al. 2007, 2009). This wide distribution was attributed to drainage rearrangements through river capture events as well as palaeoriver systems during the last glacial maximum (LGM) about 18000 years ago, when the coastline was about -130 m below the current level (Tankard 1976; Rogers 1985; Ramsay and Cooper 2002; Compton 2011; Cawthra et al. 2014), thus facilitating confluence of adjacent rivers before reaching the LGM coastline (Swartz et al. 2007, 2009). Swartz et al. (2007), however, identified four haplogroups with strong geographic affinities within the Forest redfin based on mtDNA Control Region sequences. These four haplogroups, differentiated by 0.9–2.3% genetic distance, are restricted to the Klein Brak River system, the Wilderness Lakes region, rivers discharging into the Plettenberg Bay area, and the Tsitsikamma River system, have no haplotypes shared among them (Swartz et al. 2007). In the present study, additional non-type specimens from Keurbooms and Tsitsikamma were also examined. The Keurbooms, a river system in the Plettenberg Bay area, represents a central locality within the distribution range of the Forest redfin. The Tsitsikamma on the other hand, represents the easternmost locality which also corresponds to the third haplogroup. Thus, specimens from three main geographic areas representing three of the four haplogroups were morphologically examined in the present study. Careful examination of specimens from these haplogroups showed no clear morphological differences and are therefore herein considered to represent a single species, *P.outeniqua*. Nevertheless, further examination of additional characters in freshly collected specimens is required to ascertain whether there could be potential distinguishing features (e.g., live colour pattern) that could consistently separate these haplogroups. This is the next phase of the ongoing work being undertaken by researchers at NRF-SAIAB.

The new species needs to be viewed as comprising four Management Units (MUs; Moritz 1994), each corresponding to one of the four allopatric haplogroups identified by Swartz et al. (2007). Species structured into MUs (i.e., autonomous populations), necessitate separate monitoring and management of each unit. Therefore, if one MU (e.g., Klein Brak) is overexploited or extirpated by humans or other causes, it cannot be recovered by moving fish from the Wilderness Lakes region, the Plettenberg Bay area or the Tsitsikamma River system, because they represent different MUs/haplogroups. The four MUs are each subject to different and distinct threats stemming from differences between the area occupied by each haplogroup and levels of habitat degradation within these areas; thus, they are natural candidates for differentially optimised management strategies. Dedicated surveys are also required to determine the status of each of these MUs and identify appropriate management interventions as this region is impacted by a number of environmental pressures, including forestry activities, presence of non-native species, hydrological modifications, and pollution.

### ﻿Other material examined for morphology

*Pseudobarbusafer*: • SAIAB 34422, 5 males (44.9–65.5 mm SL), 5 females (59.2–74.5 mm SL), Blindekloof River, Groendal Wilderness, Swartkops River system, South Africa, -33.68330001, 25.2999992371, collected by D. Boulle and P.H. Skelton, 11 November 1988. • SAIAB 34428, 5 unsexed, 60.1–75.1 mm SL, Blindekloof River, Groendal Wilderness, Swartkops River system, South Africa, -33.68330001, 25.2999992371, collected by D. Boulle, 8 June 1989. • SAIAB 121688 (formerly AMG 2524), 24 unsexed, 46.0–81.0 mm SL, Elands River, Swartkops River system, South Africa, -33.7667, 25.1278, collected by P.H. Skelton and A. Bok, 5 September 1974. • SAIAB 119909 (formerly AMG745), 5 unsexed, 46.0–61.0 mm SL, Elands River, Swartkops River system, South Africa, -33.71667, 25.1, collected by R.A. Jubb, 15 February 1964; • SAIAB 119773 (formerly AMG 609), 30 unsexed, 48.5–66.5 mm SL, Wit River, Sundays River system, South Africa, -33.33, 25.683, collected by R.A. Jubb, 8 April 1959. • SAIAB 119940 (formerly AMP 776), 5 unsexed, 43.0–82.0 mm SL, Kragga Kamma, Baakens River system, South Africa, -33.95, 25.5, collected by D. Bicknell, 15 January 1964.

*Pseudobarbusasper*: • SAIAB 120908, 5 unsexed, 47.8–50.2 mm SL, Groot at Steytlerville, Gamtoos River system, South Africa, -33.375, 24.35, collected by P.H. Skelton, 18 June 1972. • SAIAB 122229, 3 unsexed, 47.5–53.0 mm SL, Groot, Gamtoos River system, South Africa, -33.475, 24.6972, collected by A.H. Bok, P.H. Skelton and J. Stephenson, 18 July 1975. • SAIAB 131821, 5 unsexed, 34.0–58.0 mm SL, Le Roux, Grobelaars, Gourits River system, South Africa, -33.42472, 22.25416, collected by S. Thorne, 26 February 1988. • SAIAB 128368, 11 unsexed, 30.0–77.0 mm SL, Klein le Roux, Gourits River system, South Africa, -33.40861, 22.29138, collected by M. Brett and S. Thorne, 26 February 1982. • SAIAB 128359, 41 unsexed, 10.5–73.0 mm SL, Grobelaars, Gourits River system, South Africa, -33.41305, 22.2438, collected by M. Brett and S. Thorne, 25 February 1982. • SAIAB 130256, 20 unsexed, 23.0–55.0 mm SL, Olifants, Gourits River system, South Africa, -33.61861, 22.236, collected by S. Thorne, 13 March 1986. • SAIAB 128449, 2 unsexed, 52.5–79.5 mm SL, Grobelaars, Olifants, Gourits River system, South Africa, -33.48027, 22.2461, collected by S. Thorne and M. de Klerk, 11 December 1982. • SAIAB 128447, 16 unsexed, 26.0–57.5 mm SL, Grobelaars, Olifants, Gourits River system, South Africa, -33.53972, 22.24027, collected by S. Thorne and M. de Klerk, 11 December 1982. • SAIAB 131823, 20 unsexed, 24.0–70.0 mm SL, Grobbelaars, Olifants, Gourits River system, South Africa, -33.41305, 22.24583, collected by S. Thorne, 26 February 1988. • SAIAB 130240, 11 unsexed, 24.0–58.0 mm SL, Olifants, Gamka, Gourits River system, South Africa, -33.65138, 22.1616 mm SL, collected by S. Thorne, 13 March 1986. • SAIAB 60517, 17 unsexed, 19.4–39.5 mm SL, Grobbelaars, Gourits River system, South Africa, -33.61420059, 22.2099990845, collected by C. McKie and M. Scott, March 1999. • SAIAB 60490, 13 unsexed, 35.1–65.4 mm SL, Grobbelaars, Gourits River system, South Africa, -33.55279922, 22.2275009155, collected by C. McKie and M. Scott, March 1999. • SAIAB 59673, 29 unsexed, 25.6–66.7 mm SL, Grobbelaars, Gourits River system, South Africa, -33.39670181, 22.2150001526, collected by R. Bills and S. Mangold, 26 October 1998.

*Pseudobarbuskubhekai*: • SAIAB 204589, holotype, male, 60.5 mm SL, Umzimkhulu River system (exact locality not indicated due to conservation sensitivities), collected by A. Chakona, N. Mazungula, S. Kubheka, and N. Ntuli, 25 May 2017. • SAIAB 246079, paratypes, 9 unsexed, 45.9–62.9 mm SL, same locality information and collectors as SAIAB 204589. • SAIAB 246080, paratypes, 3 unsexed, 47.2–78.3 mm SL, same locality information as holotype, collected by P.S. Kubheka and N.S. Ntuli, 26 May 2017.

*Pseudobarbusphlegethon*: • SAIAB 51367, 9 unsexed, 51.7–55.7 mm SL, 3–4 km downstream from Algeria, Rondegat River, Olifants System, South Africa, -32.34999847, 19.0333003998, collected by R. Bills, D. Impson and M. Marriott, 11 March 1996. • SAIAB 75826, 5 unsexed, 53.1–60.2 mm SL, Rondegat River, Olifants System, South Africa, -32.37333297, 19.0602779388, collected by R. Bills, 18 April 2005. • SAIAB 75783, 3 unsexed, 49.7–58.6 mm SL, Algeria below weir, Rondegat River, Olifants System, South Africa, -32.37333297, 19.0602779388, collected by R. Bills, 13 September 2004. • SAIAB 58324, 5 unsexed, 54.4–61.8 mm SL, below forestry camp, Rondegat River, Olifants System, South Africa, -32.35139846, 19.0333003998, collected by R. Bills and D. Naran, 05 February 1998.

*Pseudobarbusquathlambae*: • SAIAB 189215, 3 unsexed, 31.5–49.8 mm SL, Himeville, Natal, Mkhomazi River system, South Africa, -29.72078, 29.51226, collected by M. Copeland, unknown date. • SAIAB 131399, 7 unsexed, 54.0–76.4 mm SL, Jordane River, Lesotho, -29.432, 28.07805, collected by K.J. Meyer, 26 May 1986. • SAIAB 25491, 4 unsexed, 59.5–76.1 mm SL, Jordane River, Lesotho, -29.39500045, 28.0424995422, collected by K.J. Meyer, 27 October 1985. • SAIAB 131400, 4 unsexed, 69.1–86.4 mm SL, Bokong River, Lesotho, -29.27027, 28.126, collected by K.J. Meyer, 24 May 1986. • SAIAB 29001, 6 unsexed, 59.3–79.5 mm SL, Sani River, Lesotho, -29.56083297, 29.2652778625, collected by P.H. Skelton, 22 September 1988. • SAIAB 63417, 4 unsexed, 61.6–73.3 mm SL, Tsoelikane Falls, Tsoelikana River, Orange System, Lesotho, -29.89749908, 29.1205997467, collected by R. Bills & J. Rall, 02 October 2000. • SAIAB 63409, 2 unsexed, 76.1–76.5 mm SL, headwaters of Moremoholo River, Orange System, Lesotho, -29.12470054, 29.3271999359, collected by R. Bills & J. Rall, 29 September 2000. • SAIAB 63408, 2 unsexed, 65.6–65.7 mm SL, Senqu River, Orange System, Lesotho, -28.92280006, 29.0242004395, collected by R. Bills & J. Rall, 29 September 2000. • SAIAB 63408, 2 unsexed, 81.1–90.4 mm SL, Matsoku River, Orange System, Lesotho, -29.2838993, 28.5531005859, collected by M. Nthimo, 07 February 2000.

*Pseudobarbussenticeps*: • SAIAB 304 (holotype), male, 65.7 mm SL, Assegaaibosch River, Krom River system, South Africa, -33.91669845, 24.3332996368. • SAIAB 200302, 9 unsexed, 23–83 mm SL, Assegaaibos River, Krom River system, South Africa, -33.9452778, 24.3139167, collected by R. Bills, V. Bills and D. Naran, 12 August 2014. • SAIAB 121815 (formerly AMG 2651), 29 unsexed, 45–75 mm SL, Assegaaibosch River, Krom River system, South Africa, -33.9413889, 24.3188889, collected by P.H. Skelton and J. Stephenson, 20 January 1975.

*Pseudobarbusswartzi*: • SAIAB 203792 (holotype), male, 80.9 mm SL, tributary of the Wabooms, Gamtoos River system, South Africa, -33.8639772, 23.8263333, collected by A. Chakona, B. Motshegoa, N. Mazungula, W. Kadye and R. Smith, 21 January 2015. • SAIAB 203793, 9 unsexed, 35.4–76.0 mm SL, Tributary of the Wabooms, Gamtoos River system, South Africa, -33.8639772, 23.8263333, collected by A. Chakona, B. Motshegoa, N. Mazungula, W. Kadye and R. Smith, 21 January 2015. • MARC 2016-032-P-0001-0004, 4 unsexed, 50.2–61.4 mm SL, main tributary of the Louterwater River, Gamtoos River system, South Africa, -33.8333611, 23.6373056, collected by A. Chakona, S. Reddy and R. Smith, 18 January 2016. • SAIAB 203772, 10 unsexed, 25.5–57.9 mm SL, western tributary of the Louterwater River, Gamtoos River system, South Africa, -33.82575, 23.631, collected by A. Chakona, S. Reddy and R. Smith, 18 January 2016. • SAIAB 203779, 6 unsexed, 32–64.8 mm SL, Main Tributary of the Louterwater River, Gamtoos River system, South Africa, -33.8333611, 23.6373056, same collectors and date as SAIAB 203772. • SAIAB 203787, 34 unsexed, 18.2–86.7 mm SL, upper Dwars River, Gamtoos River system, South Africa, -33.6534444, 23.7539722, same collectors and date as SAIAB 203772. • SAIAB 203786, 17 unsexed, 34.8–64.9 mm SL, Klein River at Kouga Wilderness, Gamtoos River system, South Africa, -33.7112222, 23.8440833, same collectors as SAIAB 203772, 19 January 2016. • SAIAB 203789, 8 unsexed, 47.8–70.2 mm SL, Braam River, Gamtoos River system, South Africa, -33.7135278, 23.8465833, same collectors as SAIAB 203772, 19 January 2016. • SAIAB 203788, 13 unsexed, 17.9–63.3 mm SL, Diep River, Gamtoos River system, South Africa, -33.7541944, 24.0812500, collected by A. Chakona and R. Smith, 20 January 2016. • SAIAB 203781, 45 unsexed, 14.7–53.8 mm SL, upper Kansenkei River, Gamtoos River system, -33.7296667, 24.5545833, same date and collectors as SAIAB 203788. • SAIAB 203774, 10 unsexed, 25.5–57.9 mm SL, Wit River, Gamtoos River system, South Africa, -33.6538333, 24.51605556, collected by A. Chakona and B. Motshegoa, 7 March 2016. • SAIAB 203780, 5 unsexed, 24.6–58.8 mm SL, Lourie River, Gamtoos River system, South Africa, -33.8506944, 25.0388194, collected by A. Chakona and B. Motshegoa, 7 March 2016. • SAIAB 120538 (formerly AMG1374), 70 unsexed, Kouga Dam, Gamtoos River system, South Africa, -33.6666667, 24.5166667, collected by F. Farquharson, 6 July 1967. • SAIAB 120539, 70 unsexed, same locality and collector as SAIAB 120538.

## Supplementary Material

XML Treatment for Pseudobarbus outeniqua

## ﻿Additional information

### Author ORCIDs

Fatah Zarei https://orcid.org/0000-0001-5552-4301

Pedro H. N. Bragança https://orcid.org/0000-0002-8357-7010

Paul H. Skelton https://orcid.org/0000-0001-9587-2802

Albert Chakona https://orcid.org/0000-0001-6844-7501

### Data availability

All of the data that support the findings of this study are available in the main text.
